# Optimizing Electrode Configurations for Wearable EEG Seizure Detection Using Machine Learning

**DOI:** 10.3390/s23135805

**Published:** 2023-06-21

**Authors:** Hagar Gelbard-Sagiv, Snir Pardo, Nir Getter, Miriam Guendelman, Felix Benninger, Dror Kraus, Oren Shriki, Shay Ben-Sasson

**Affiliations:** 1 NeuroHelp Ltd., Ramat-Gan 5252181, Israel; hagarg@neuro-help.com (H.G.-S.); snirp@neuro-help.com (S.P.); nirg@neuro-help.com (N.G.); miriamg@neuro-help.com (M.G.); shayb@neuro-help.com (S.B.-S.); 2 Department of Cognitive and Brain Sciences, Ben-Gurion University of the Negev, Beer-Sheva 8410501, Israel; 3 Department of Neurology, Rabin Medical Center, Beilinson Hospital, Petach Tikva 4941492, Israel; felixbenninger@gmail.com; 4 Sackler Faculty of Medicine, Tel Aviv University, Tel Aviv 6997801, Israel; drorkrausmd@gmail.com; 5 Department of Pediatric Neurology, Schneider Children’s Medical Center of Israel, Petach Tikva 4920235, Israel

**Keywords:** seizure detection, wearable EEG, machine learning, continuous EEG monitoring, electrode configuration optimization, computational efficient, metric adjustment

## Abstract

Epilepsy, a prevalent neurological disorder, profoundly affects patients’ quality of life due to the unpredictable nature of seizures. The development of a reliable and user-friendly wearable EEG system capable of detecting and predicting seizures has the potential to revolutionize epilepsy care. However, optimizing electrode configurations for such systems, which is crucial for balancing accuracy and practicality, remains to be explored. This study addresses this gap by developing a systematic approach to optimize electrode configurations for a seizure detection machine-learning algorithm. Our approach was applied to an extensive database of prolonged annotated EEG recordings from 158 epilepsy patients. Multiple electrode configurations ranging from one to eighteen were assessed to determine the optimal number of electrodes. Results indicated that the performance was initially maintained as the number of electrodes decreased, but a drop in performance was found to have occurred at around eight electrodes. Subsequently, a comprehensive analysis of all eight-electrode configurations was conducted using a computationally intensive workflow to identify the optimal configurations. This approach can inform the mechanical design process of an EEG system that balances seizure detection accuracy with the ease of use and portability. Additionally, this framework holds potential for optimizing hardware in other machine learning applications. The study presents a significant step towards the development of an efficient wearable EEG system for seizure detection.

## 1. Introduction

### 1.1. Advancements and Limitations of Wearable Systems for Seizure Detection

Epilepsy is a neurological disorder that affects nearly 1% of the world population, and is characterized by recurrent seizures [1,2]. These seizures can be life-threatening [3,4], and have a significant impact on the quality of life of those who are affected. While most people with epilepsy (PWE) can achieve seizure control with antiepileptic drugs (AEDs), approximately one-third of PWE suffer from drug-resistant epilepsy (DRE), which does not respond well to medical treatment [5]. Despite optimal drug therapy, these individuals experience recurrent seizures and often require more invasive treatments, such as surgery [6,7], implantable devices [8], or dietary modifications [9]. Additionally, even patients with drug-controlled epilepsy may still experience occasional seizures, with approximately 56% of adult PWE experiencing seizures overall [10].The unpredictability of seizures, particularly in people with DRE, constitutes one of the main challenges that impacts the quality of life of PWE [11,12]. Accurate and timely seizure detection could enable interventions and treatment monitoring that have the potential to improve the quality of life for PWE. In particular, this could help with the optimization of the medical treatment given to patients with epilepsy, including medication adjustments, monitoring of medication efficacy [13], evaluating seizure-prevention strategies, and generating alerts for required emergency interventions [14].

One promising avenue for improving the quality of life of patients suffering from unpredictable seizures is through the use of wearable systems, which can detect seizures in real-time and alert patients or caregivers to take the appropriate action [15]. Multiple products to detect seizures are commercially available; these are based on movement detectors, audio, autonomic change detection (such as skin conductance [16]), and electroencephalography (EEG) [17,18,19,20,21]. However, peer-reviewed performance reports have been published for only a small number of these devices. In most cases, these focus on the detection of generalized tonic-clonic seizures or focal to bilateral tonic-clonic seizures [20,22]. The focus placed on this type of seizure is due to their robust clinical and behavioral correlates, which manifest in the wide range of modalities used [23]. However, many patients exhibit different types of seizures, including absence seizures or focal aware seizures. These seizures may have only a mild clinical manifestation that will not be detected in every peripheral modality, yet it may be identified in the EEG recording [23]. Some of these seizures may have a widespread electrographic manifestation, e.g., absence seizures, whereas some seizures may manifest in a local subset of EEG electrodes, e.g., focal seizures. Thus, in the process of creating a wearable EEG-based seizure monitoring system, it is essential to identify the critical recording locations that should be included in order to ensure adequate seizure detection performance in a wide variety of patients and epileptogenic foci. 

The potential advantages of an ambulatory wearable EEG system extend beyond its ability to detect various seizure types with a higher sensitivity. Such a system allows for the continuous long-term monitoring of brain electrophysiological activity, which can provide useful insights into the long-term dynamics of the signal, and aid in characterizing neuronal changes that influence susceptibility to seizures over extended periods during both wakefulness and sleep [24,25,26]. Given the high comorbidity between epilepsy and sleep disorders [27,28], a practical wearable EEG system could therefore help quantify the relationship between seizures and sleep, leading to more accurate detection and better understanding of epilepsy classification in the short-term [29]. Over the long-term, measuring sleep quality and epileptiform activity during sleep may have a predictive value in determining the likelihood of seizures occurring in the following day [29].

### 1.2. Balancing Machine Learning Performance and Ergonomics in Wearable EEG Systems

The development of an accurate, comfortable, and user-friendly wearable EEG system for brain activity monitoring and, in particular, for the early detection of epileptic seizures is a challenging task [15,30]. It requires the careful balancing of competing priorities: on the one hand, the system must provide high-quality signals and be highly accurate in detecting seizures. On the other hand, it must be easy to set up and use for prolonged periods of time [15,31,32]. 

Achieving this balance requires a precise definition of performance measures for the detection algorithm. First, the system must be sensitive and maximize the proportion of identified seizures, typically termed as sensitivity or recall. On the other hand, the system must produce a minimal false alarm rate. A high frequency of false alarms may deem the system alerts as a nuisance, and significantly reduce the compliance and alert value as a result. Typically, algorithmic performance is evaluated using the area under the receiver operating curve (AUC-ROC), where a perfect classifier would produce a score of one. The curve is created by plotting the sensitivity or true-positive rate (TPR) against the specificity or false-positive rate (FPR), which, in our case, is the proportion of false alarms out of all the non-seizure data, along the 0–1 decision threshold range. 

In the context of seizure detection, the proportion of true labels is extremely low, leading to substantially imbalanced data. In imbalanced datasets, the AUC ROC may give an overly optimistic estimate of the model’s performance. When the positive class is rare, the model can achieve a high TNR by simply predicting all samples as negative, resulting in a high specificity, but also accompanied with a low sensitivity. In terms of false alarm evaluation, a relatively low FPR may still be an order of magnitude higher than the actual seizure prevalence, thereby creating more false alarms compared to the true-positive seizure alerts and giving a low predictive value to an alert. A complementary metric to use for evaluating the predictive value of the alerts is the positive predictive value (PPV), also known as precision, which is calculated by evaluating the proportion of true alerts out of all the alerts, hence providing information on the alert value. 

**Figure 1:**
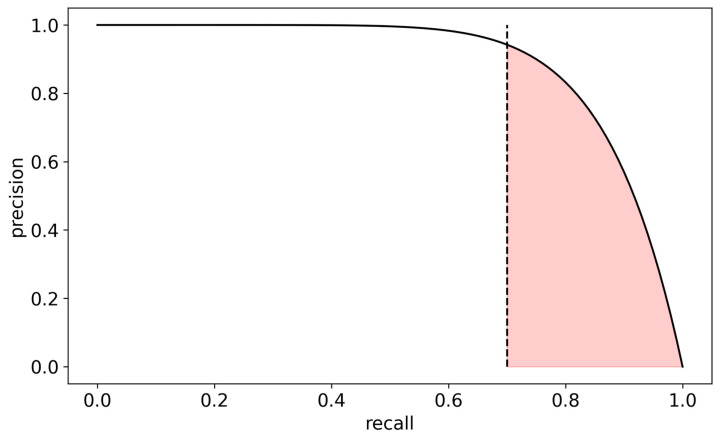
Definition of the AUC-PR-0.7 performance measure—the area (marked in red) under the precision–recall curve above 70% recall (dashed line). Precision is defined as the relative number of true alerts out of all alerts, while recall is defined as the relative number of alerted seizures out of all seizures.

Both measures of sensitivity (recall) and PPV (precision) are considered in the precision–recall (PR) curve (Figure 1). The PR curve focuses on the positive class and plots the precision against recall along the 0–1 decision threshold range. The PR curve provides a more accurate measure of the model’s ability to identify positive samples in imbalanced datasets accurately, and it is more appropriate when the positive class is rare [33]. To further adjust this measure to our problem, we decided to focus on the area under the curve using only clinically acceptable sensitivity ranges (>0.7, i.e., more than 70% detected seizures), hence defining our target performance metric as the AUC-PR-0.7 (Figure 1), and thereby giving us the ability to specifically optimize alert values at the high sensitivity range of interest. This measure could then be used to systematically evaluate the performance of various electrode layout options.

One crucial factor that can affect the performance of a wearable EEG system is the number and location of the electrodes [34]. While a larger number of electrodes can potentially provide more information and improve the accuracy of seizure detection, it also increases the complexity and cost of the system as a result. One way of reducing this complexity is by identifying the optimal subsets of electrodes that maximize the performance while minimizing the complexity of the mechanical design. When evaluating an algorithm for cross-subject performance, we must take into account patient variations in the signal. These may occur due to the differences in age, gender, etiology, seizure focus, and additional factors affecting the ictal activity and the baseline non-ictal activity. Thus, the cross-subject classification framework should only rely on the essential common representations to thereby avoid overfitting patient-specific characteristics and learn the shared patterns. This should be taken into account when choosing an optimal electrode subset.

In this article, we describe a systematic approach to identifying optimal electrode configurations for a wearable EEG system for epileptic seizure detection. We first defined the performance measure to be optimized and then systematically evaluated the cross-subject performance of various electrode configurations using real-world EEG data. Our results demonstrate the trade-off between accuracy and complexity and provide valuable insights into the optimal electrode configurations for practical usage. 

Our findings can assist in the development of wearable EEG systems for epileptic seizure detection at home and in clinical settings and can ultimately improve the lives of people with epilepsy. The framework we outline here can be used also for the development of wearable EEG systems in additional domains.

The structure of the paper is as follows: Section 2 provides an overview of the analytical framework, including the data, the extracted features, the machine learning algorithm employed, the performance evaluation metrics, and the technical aspects concerning the computational implementation. In Section 3, we present the results of our analysis, initially focusing on the trade-off between accuracy and system complexity. We then delve into a systematic examination of all eight-electrode configurations. Section 4 discusses the implications of our findings, offering insights into the practical application of optimal electrode configurations and suggesting avenues for future research. Finally, in Section 5, we summarize the key contributions of this work towards the development of an efficient and user-friendly wearable EEG system for the detection of epileptic seizures.

## 2. Materials and Methods

### 2.1. Data

We used the EPILEPSIAE database [35], a large EEG dataset that was recorded in different epilepsy centers across Portugal (Coimbra), France (Paris), and Germany (Freiburg) [35], where the recorded patients were undergoing pre-surgical evaluation [5]. The criteria defined for this dataset stated that the data for each patient must include at least 69 h (4 days) and at least five clinical epileptic seizures with interictal intervals of at least 4 h between each other [35]. 

This database includes 24,969 h of EEG recordings from the inpatient pre-surgical evaluation of 158 people with focal epilepsy (92 males; age 34.4 ± 12.3; 158.0 ± 85.7 recording hours per patient; and 7.7 ± 4.8 seizures per patient). In total, 75% had presumed temporal lobe epilepsy, and 12% had presumed frontal lobe epilepsy, respectively. EEG recordings included 26–41 electrodes (31.0 ± 2.6 mean ± standard deviation). The 19 electrodes of the international 10–20 system (FP1, FP2, F7, F3, Fz, F4, F8, T3, C3, Cz, C4, T4, T5, P3, Pz, P4, T6, O1, and O2, respectively) were included in all recordings, and were thus used in our analysis. The dataset also includes the medical history and seizure types. Periods that contained epileptic seizures were annotated by two experienced independent reviewers. Seizure annotations included onset and offset times, vigilance state, and seizure classification. Note that since the dataset only included patients with focal epilepsy, all seizures in the dataset have focal onset. The EPILEPSIAE database has been widely used in research studies to develop and assess algorithms for seizure detection and prediction, as well as to explore other aspects of epilepsy, such as brain connectivity and network dynamics [36]. 

### 2.2. Preprocessing and Feature Extraction

EEG data collected from the sensors were referenced to the electrode FP2. The choice of electrode FP2 was motivated by its convenient location on the forehead, allowing for a good conductance and attachment and, therefore, a reliable signal. Furthermore, a preliminary examination conducted with an average reference demonstrated that the combined contributions of both FP1 and FP2 electrodes are equivalent to the contribution made by each of these electrodes individually. Using FP2 as the reference left a total of 18 electrodes for the analyses. The data were bandpass filtered in the range of 1–40 Hz, and a notch filter at 50 Hz was applied to remove the line noise from the electricity network. We employed a group of automated criteria to eliminate sections of the data that exhibited a high degree of noise or interference or contained a non-physiological dominant component in the signal. The data were divided into 20 s segments, with a 10 s overlap between consecutive segments. Segments that partially or fully overlapped a seizure according to the database annotations were labeled as ictal, and all other segments were labeled as non-ictal.

For the analysis in this study, we used 17 families of spectral single-channel EEG features extracted for each 20 s segment. Specifically, for each channel, we calculated the following [17]:

- Broadband root total power (the square root of the power integral, which is equivalent to the temporal signal’s standard deviation);
- The relative power and relative log power in 5 frequency bands: delta [1–4 Hz], theta [4–8 Hz], alpha [8–12 Hz], beta [12–30 Hz], and gamma [30–50 Hz]. The term ‘relative’ indicates that the power in each band was divided by the total broadband power between 1–40 Hz;
- Spectral moment: first, the signal’s power distribution in the frequency domain is normalized as a probability distribution, followed by computation of the expected power value;
- Spectral edge frequency (SEF): the frequency below which 90% of the total EEG power is located;
- Spectral entropy (SE): the entropy of the spectrum when treated as a normalized probability distribution;
- Spectral slope and intercept: the slope and intercept of the linear of the log power log frequency plot;
- “Out-of-range” feature: This feature counts how many of the following four features are out of these features’ normal empirically defined range: the root total power of the power spectrum, the spectral slope and intercept, and the spectral edge frequency. Thus, this feature is an integer number between 0 and 4.

### 2.3. Machine Learning Algorithm

We used the LightGBM (light gradient-boosting machine) machine learning algorithm for the analysis and classification of EEG data [31]. LightGBM is a popular and highly efficient gradient-boosting framework that uses decision tree-based algorithms to iteratively improve the accuracy of predictions. The algorithm optimizes the cross-entropy loss, a commonly used loss function in binary classification problems: $L=-\frac{1}{N}\sum_{n=1}^{N}\left[y_{n}\log\left({\hat{y}}_{n}\right)+\left(1-y_{n}\right)\log\left(1-{\hat{y}}_{n}\right)\right]$, where N is the number of samples, $y_{n}$ is the true label, and ${\hat{y}}_{n}$ is the predicted label. LightGBM was designed to handle medium-scale datasets and provides faster training and prediction speeds compared to other machine learning algorithms. Previous analyses performed with an ensemble of different algorithms (support vector machine, linear discriminant analysis, extra trees, logistic regression, and random forest) have shown that the performance of the ensemble was only negligibly better than that of the LightGBM. Hence, LightGBM was selected for the current analyses.

### 2.4. Data Splitting into Train and Test Sets

Data were split into a train set and a test set at the level of patients. The training set contained balanced data (the number of ictal and non-ictal periods was equal) from 75% of the patients (n = 120), while the test set included all data (not balanced) from the remaining 25% of the patients, respectively (n = 38). While splitting the data at the patient level is more challenging for the model, this approach ensures the model’s generalizability and its applicability in real-life scenarios, where it needs to perform well with patients it has never encountered before. We used 20 different train-test splits of the data. All train-test data splits preserved the frequency of the epileptic focus zones in the full dataset in terms of the seizure onset zone (temporal, frontal, parietal, or occipital) and hemisphere (right or left). Implementing this procedure was crucial in diminishing the susceptibility of the findings to a specific division of the data and enhancing their generalizability.

Hyper-parameter optimization was only performed once—on the training set that included the full electrode set (n = 18 electrodes) and the first split of data. These hyper-parameters were used for all models that were built in the rest of the analyses.

### 2.5. Performance Measure

An important measure of performance in binary classification on imbalanced data is the area under the precision–recall curve (AUC-PR). The precision–recall curve depicts the precision (or PPV) of the algorithm, namely the relative number of true alerts out of all alerts, as a function of the recall (or sensitivity), namely the relative number of alerted seizures out of all seizures. As a low sensitivity is irrelevant for a viable seizure detection algorithm, we only focused on the area under the precision–recall curve in the recall range of 0.7–1 (Figure 1) and normalized it to the 0–1 range, i.e., $\frac{1}{0.3}\;\overset{1}{\underset{0.7}{\int }}p\left(r\right)dr$; where $p\left(r\right)$ is the precision $\left(p\right)$ as a function of recall $\left(r\right)$. We denoted this quantity as AUC-PR-0.7. We empirically found that this quantity is a good proxy for several other performance measures of interest which are more computationally intensive (e.g., the mean number of false alarms per day across patients or mean total false alarm time per day across patients). Therefore, this proxy measure was used for model optimization. In addition, we also used the more traditional metrics of the area under the receiver operating characteristic curve (AUC-ROC).

### 2.6. Analytical Framework

The analytical framework was divided into two successive stages. The goal of the first stage was to determine the optimal number of electrodes, while the goal of the second stage was to find the optimal configurations with this number of electrodes. 

**Figure 2:**
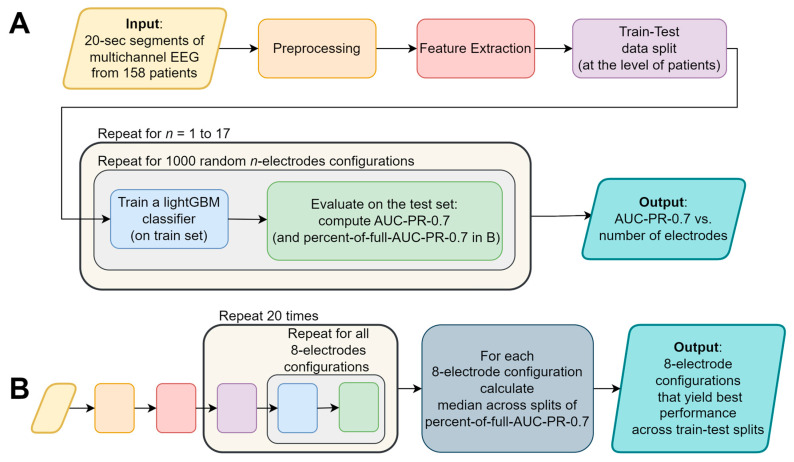
A flowchart depicting the major stages of the analysis: (**A**) determining the optimal number of electrodes, and the (**B**) systematic examination of all eight-electrode configurations. The colors of the flowchart symbols represent the processing stage; the text of identical processing stages was omitted from (**B**) for conciseness.

To determine the optimal number of electrodes we sampled 1000 random configurations of each possible number of electrodes (from 1 to 17 out of 18 electrodes; there are less than 1000 possible configurations of 1–3 and 15–17 electrodes, hence in these cases we sampled all possible configurations). For each configuration we built a LightGBM model and evaluated its performance using the AUC-PR-0.7 measure. We compared the sampled configurations AUC-PR-0.7 to that of the full model containing all 18 electrodes. In this analysis stage, a single data split was used (as shown in Figure 2A).

After determining the optimal number of electrodes (eight electrodes, not including the ground and reference; as shown in Section 3), we examined the performance of the algorithm across all possible eight-electrode configurations (43,758 options; $\left(\begin{array}{c} n\\ k \end{array}\right)=\frac{n!}{k!\left(n-k\right)!}\;for\;n=18;\;k=8$). To that end, we built a LightGBM model for each of the configurations and evaluated its performance using the AUC-PR-0.7 measure. We repeated this for each of the 20 data splits. To generalize the results across these data splits, we calculated for each electrode configuration in each data split the ratio between the AUC-PR-0.7 of a model using that configuration’s electrodes only, and the AUC-PR-0.7 of the corresponding full model (containing all 18 electrodes and all features). We denoted this value as “percent-of-full-AUC-PR-0.7” and calculated the median percent-of-full-AUC-PR-0.7 across the data splits (see Figure 2B). 

### 2.7. Runs and Computational Resources

We used an AWS ECS cluster to extract features from 20 s epochs of EEG data for each of the 158 patients. Subsequently, we created 20 train-test splits of these patients’ features. Balanced train sets consisted of 8000–14,000 samples (depending on the number of seizure segments in each train set), while test sets consisted of ~1.5–2 million samples (depending on the recording duration of patients in each test set). To examine the case of eight electrodes, we conducted an exhaustive search on all possible 43,758 configurations of eight electrodes out of 18 (all 10–20 system electrodes, besides FP2, which was used as a reference). Using another AWS ECS cluster, we trained and evaluated a LightGBM binary classifier for each of the 43,758 electrode configurations using the 20 different train-test splits, resulting in a total of 875,160 models. The performance of each model was then evaluated on its corresponding test set using the AUC-PR-0.7 and AUC-ROC metrics. 

As the test sets were big in size (~2 million samples), the inference phase turned out to be the slowest link in the training-evaluation chain. In order to accelerate the inference phase, we used the Intel extension for Scikit-learn (https://github.com/intel/scikit-learn-intelex, accessed on 17 November 2022), and achieved a four-fold decrease in the computation time compared to the vanilla LightGBM implementation. To further cut running times, we only used five selected feature families in this analysis (relative power in delta, theta, and alpha bands, broadband root total power, and the number of features out-of-range, see Section 2.2). This feature set was selected based on domain knowledge and yielded a similar performance to the full feature set.

## 3. Results

EEG data from 158 patients of the EPILEPSIAE database [35] were divided into 20 s segments, and were labeled as either *ictal* (during a seizure) or *non-ictal* (not during a seizure), according to the database annotations. Spectral features were extracted, and data were split to train-test sets at the level of patients. LightGBM models were trained on a balanced (ictal/non-ictal) train set and were evaluated on a full (unbalanced) test set. Performance was measured using AUC-PR-0.7 and AUC-ROC (see Section 2). 

### 3.1. Dependence of Classification Performance on Electrode Number

**Figure 3:**
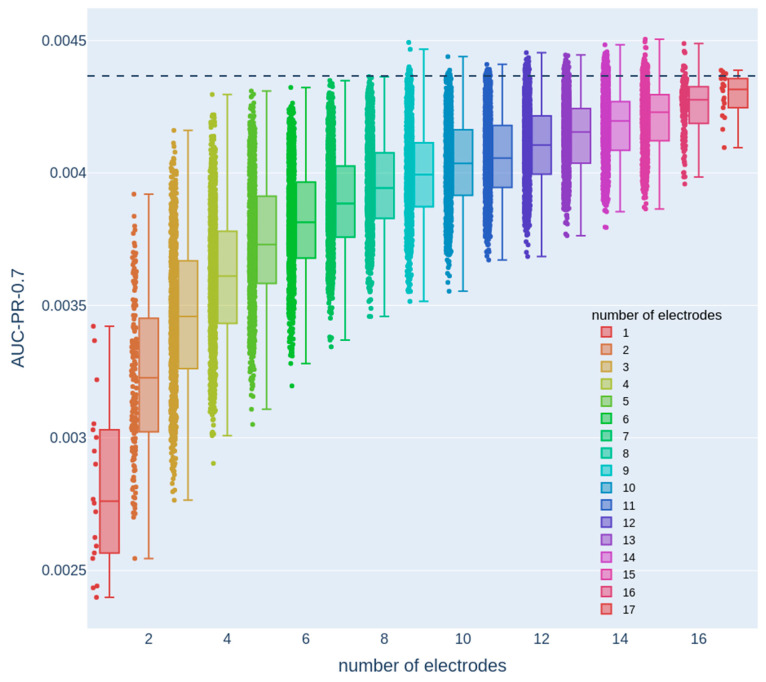
Dependence of the AUC-PR-0.7 measure on the number of electrodes. Each color denotes a different number of electrodes, and each dot represents the test set AUC-PR-0.7 of a model that only uses the features from a specific electrode configuration with the corresponding number of electrodes. The dashed line represents the AUC-PR-0.7 measure obtained from a model that uses features from all 18 electrodes.

Our objective was to strike a balance between the system usability and performance by minimizing the number of electrodes while maintaining comparable detection capabilities to those achieved with the full set of electrodes. This approach aimed to enhance user-friendliness, thereby ensuring that the wearable EEG system remains practical and accessible without compromising its overall performance. With that objective in mind, we first examined the performance as a function of the number of electrodes (see Figure 2A for an analysis summary flowchart). To that end, we examined 1000 random configurations for each possible electrode-subset size (one to seventeen electrodes; Figure 3) and compared it to the performance of the full set containing all 18 electrodes (black dashed line). 

Here, we used a single train-test data split. This analysis revealed that there were numerous configurations of eight electrodes with performance that was similar to the full 18-electrode 10–20 configuration, thereby allowing the mechanical design process to maintain a considerable degree of flexibility and freedom in choosing electrode configurations without compromising performance. Therefore, we next focused on systematically examining eight-electrode configurations. The decline in performance around eight electrodes was characterized with a gradual rather than sharp drop. This observation is consistent with the fact that different EEG electrodes cover overlapping areas, with each electrode encompassing a significant region of cortical activity. Consequently, when the number of electrodes is reduced to around eight, certain configurations may still provide adequate coverage, enabling the detection of relevant dynamical changes associated with epileptic seizures. However, other configurations may lack sufficient coverage, resulting in the omission of crucial seizure-related patterns.

### 3.2. Systematic Examination of All Eight-Electrode Configurations

To examine the case of eight electrodes, we conducted an exhaustive search on all possible 43,758 configurations of eight electrodes out of eighteen (see Section 2.6). For each configuration, we trained and evaluated a LightGBM binary classifier using 20 different train-test splits, resulting in a total of 875,160 models (see Figure 2B for an analysis summary flowchart). We used an AWS ECS cluster to perform this analysis in a reasonable amount of time.

**Figure 4:**
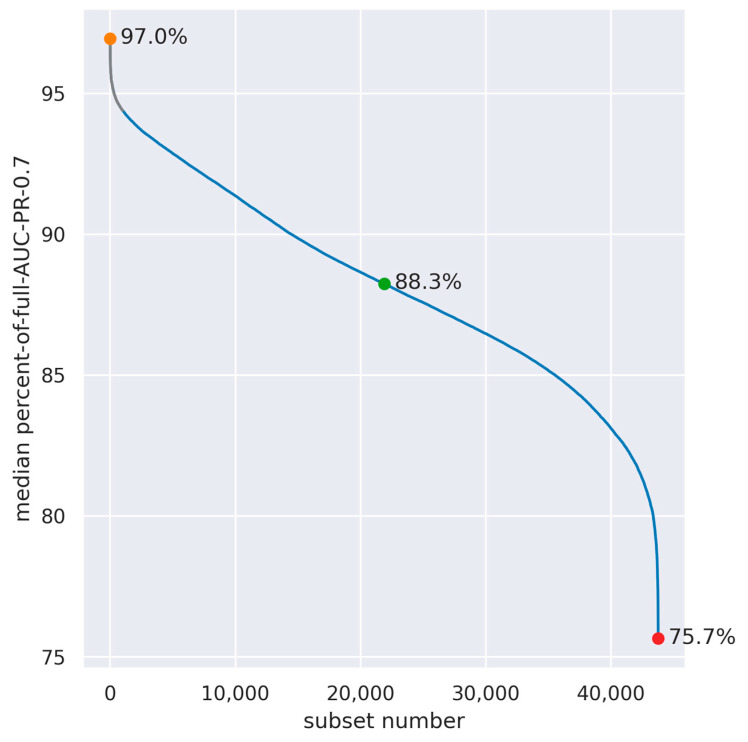
Median across data splits of the percent-of-full-AUC-PR-0.7 in all combinations of eight out of eighteen electrodes (n = 43,758). The subsets with the highest, median, and lowest median-percent-of-full-AUC-PR-0.7 are denoted by colored dots, and the spatial configuration of these subsets is presented in Figure 5. The top 1000 configurations are marked in gray.

The full models, using all electrodes and features, yielded somewhat different performance values for each data split: AUC-PR-0.7 values ranged between 0.0032 and 0.0056 (0.0040 ± 0.0006; mean ± standard deviation), and AUC ROC values were between 0.86 to 0.9 (0.88 ± 0.01; mean ± standard deviation). Similarly, the electrode configurations that yielded the highest AUC-PR-0.7 were somewhat different among the different data splits. Therefore, to generalize the results across data splits, we used the median across data splits percent-of-full-AUC-PR-0.7 (see Section 2.6). Figure 4 depicts this quantity for all 43,758 eight-electrode subsets. Although performance was found to be varied on a wide range of values (from 75.7% to 97%), the top 1000 spatial configurations (marked in gray) were all above 94.4%, indicating that there is still substantial flexibility in the electrode layout.

**Figure 5:**
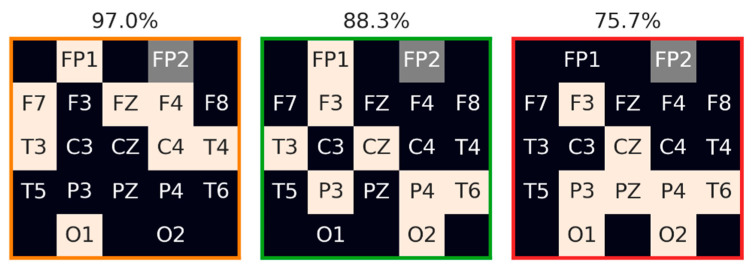
Examples of eight-electrode configurations with the highest, intermediate, and lowest median across data split percent-of-full-AUC-PR-0.7. The electrodes composing each configuration are denoted in beige. The reference electrode (FP2) is denoted in gray. Median-percent-of-full-AUC-PR-0.7 is denoted above each configuration, and the frame color is in accordance with the color of the marked data points as shown in Figure 4.

It is possible to evaluate the contribution of each electrode by examining its participation in the top subsets. However, separately examining the contribution of each electrode is an over-simplification, as it does not take into account the interactions between these electrodes; it is thus informative for the system design process to examine multiple configurations of eight electrodes, which provide a good performance, and then select the optimal configurations according to various design considerations. Figure 5 depicts three examples of these configurations—the highest, intermediate, and lowest, in terms of the median percent-of-full-AUC-PR-0.7 across the data splits.

## 4. Discussion

In recent years, there has been substantial progress made in the development of algorithms for seizure detection and prediction, primarily focusing on electrophysiological data, such as surface and intracranial EEG [37,38]. Concurrently, advancements have been made in the design and development of wearable EEG systems [39], which hold the potential to revolutionize seizure monitoring and management. However, despite the urgent need for such technology, practical and reliable EEG-based solutions that can be seamlessly integrated into the daily routines of people with epilepsy remain elusive. This gap highlights the importance of the continued research and innovation in this field, striving to develop user-friendly, accurate, and dependable wearable EEG systems that can empower patients and thereby improve their overall quality of life.

A wearable EEG system for daily use should balance performance with the ease of use and portability [39]. The system should be compact, lightweight, and unobtrusive, allowing patients to wear it comfortably for prolonged periods of time without interfering with their daily activities. In addition, the system should be easy to use, with minimal setup and maintenance requirements to reduce the burden on patients and caregivers. The design and development of such systems require careful consideration of factors, such as the electrode number and location, signal processing algorithms, and power consumption, among others [30]. Ultimately, the success of a wearable EEG system for seizure detection will not only depend on its accuracy in detecting seizures, but also on its practicality and usability in real-world settings.

In this study, our objective was to systematically examine the performance of a seizure detection algorithm under various electrode configurations in order to guide the design of a wearable EEG system and provide constraints from a machine-learning point of view. To achieve this, we utilized the EPILEPSIAE database, which allowed us to explore a large number of potential electrode configurations with variable electrode counts. Our findings demonstrated that high-performance seizure detection, comparable to that achieved with 18 electrodes, can be obtained even with configurations utilizing only eight electrodes. However, it is important to note that electrode configurations with substantially fewer electrodes might result in a low sensitivity and high false-alarm rates. Such outcomes could be detrimental to the usability of the system and patient compliance, despite the potential benefits of the smaller form factor. Therefore, it is crucial to carefully examine the different configurations in terms of both detection accuracy and various aspects of the mechanical design. 

The mechanical design of the headset should take into account multiple factors, such as usage during sleep versus wakefulness, the weight of the headset, the attachment method to the scalp, and hair penetration. For example, during sleep, patients may move around or change positions, which can cause the headset to shift or become dislodged. During wakefulness, motion-related artifacts and multiple environmental sources of noise can occur, which can be reduced by implementing the appropriate mechanical and electronic design strategies. The headset should be designed to be lightweight and comfortable, with a secure attachment mechanism that can withstand movement and minimize noise artifacts. Additionally, the design should consider hair penetration, as hair can interfere with the quality of the EEG signal, and hair-penetrating electrodes may be less convenient for the user. From a mechanical design perspective, it is desirable to minimize the number of hair-penetrating electrodes in order to enhance user comfort and improve the overall usability. 

We quantified the performance of all possible eight-electrode configurations through a comprehensive analysis. Our findings revealed that there are multiple configurations that are capable of achieving a high performance (in terms of the AUC-PR-0.7 metric), thus providing flexibility for the design process to focus on configurations that optimize user-friendliness and ergonomics. 

It is important to note that in this study, the machine learning algorithm was evaluated using a cross-patient approach, meaning it was assessed on patients who were not included in the training set. This methodology thereby ensures more robust and generalizable results. Furthermore, our study encompassed a substantial and diverse database, comprising adult patients with varying epileptic foci. Importantly, the dataset adequately represents the major types of focal epilepsy, including temporal and frontal lobe epilepsy, thereby ensuring a comprehensive coverage of the relevant cases. As a result, we have a high level of confidence in the generalizability and representativeness of the results obtained to the adult population. An additional investigation is further warranted to assess the generalizability of these findings to the pediatric population, as the EPILEPSIAE dataset utilized in our study lacks the representation of children. 

In future analyses, it may also be valuable to investigate optimal electrode configurations for specific types of epilepsy in terms of epileptic foci, etiologies, or clinical syndromes. This would require data from a sufficiently large population with the relevant type of epilepsy. However, optimizing the system for a specific population may also offer several benefits, such as reducing the mechanical complexity and improving the algorithm’s performance. By tailoring the system to individual populations, a more personalized and effective approach to seizure detection could be achieved, potentially enhancing patient outcomes and their overall quality of life.

Recently, ultra-long monitoring of EEG with minimally invasive sub-cutaneous electrodes has emerged [40], offering intriguing possibilities in the context of seizure detection [41] and sleep analysis [42]. Studies have demonstrated that the sensor signal from these sub-cutaneous electrodes exhibits a high degree of similarity to the proximate scalp sensors. We propose that an analysis similar to the one presented in this paper could further enhance the potential of ultra-long monitoring by optimizing the implantation location and the spatial arrangement of the sensors. By refining these aspects, it may therefore be possible to improve the overall effectiveness and reliability of sub-cutaneous EEG systems, and provide a more accurate and less obtrusive monitoring solution for individuals with epilepsy and other neurological conditions.

This study outlines a systematic process for optimizing the number and configuration of electrodes in an EEG system for detecting epileptic seizures in a home or ambulatory setting. By, our findings can thereby inform the development of a wearable EEG system that balances the performance and ergonomic considerations and enables early detection and timely intervention for seizures. Additional aspects, such as the type of electrodes, integration of additional physiological signals (e.g., heart rate and skin conductance), efficiency of the algorithm and preprocessing, and data storage and transmission should also be considered in order to achieve the desired balance between accuracy and usability. The use of ambulatory EEG systems could facilitate the collection of continuous data outside of clinical settings, providing a more naturalistic and comprehensive picture of a patient’s condition. While there are rich datasets available from clinical settings, the collection of ambulatory data from PWE can further enhance the field of seizure detection and prediction by providing real-world data that reflects the complexities of everyday life.

Furthermore, our approach can have broader applications in neuroscience and cognitive monitoring, enabling the identification of optimal electrode configurations for monitoring brain activity with potential prognostic value for seizure anticipation and other neurological conditions, such as Alzheimer’s disease [43] and depression [44]. Additionally, this methodology can be applied to the monitoring of cognitive workload in healthy individuals [45]. Given a labeled multi-electrode dataset in these domains, our systematic approach for recognizing optimal minimal electrode configurations can be utilized to improve the accuracy and efficiency of brain monitoring technologies, especially those designed for home-use portability. This will lead to a better diagnosis and treatment of neurological disorders, ultimately resulting in improved outcomes and well-being for individuals with various neurological conditions.

## 5. Conclusions

In conclusion, this study can serve as a meaningful step towards the development of a user-friendly, reliable, and efficient wearable EEG system for epileptic seizure detection. By systematically exploring the impact of electrode number and configuration on the performance of a seizure detection algorithm, we have demonstrated that it is possible to achieve a high accuracy with fewer electrodes and maintain a balance between ergonomics and detection performance. Our findings not only provide valuable insights for the design of wearable EEG systems tailored to the needs of people with epilepsy, but also have broader implications for other neurological conditions and cognitive monitoring applications. This research contributes to the advancement of wearable brain monitoring technologies, particularly those designed for home-use portability, thereby promoting a better diagnosis, treatment, and management of neurological disorders.

## Data Availability

Restrictions apply to the availability of these data. Data were obtained from the EPILEPSIAE dataset and are available at https://epilepsy.uni-freiburg.de/database/, with the permission of the authors [35].
